# New Insights Into Oral Squamous Cell Carcinoma: From Clinical Aspects to Molecular Tumorigenesis

**DOI:** 10.3390/ijms22052252

**Published:** 2021-02-24

**Authors:** Shang-Hung Chen, Sheng-Yen Hsiao, Kwang-Yu Chang, Jang-Yang Chang

**Affiliations:** 1 National Institute of Cancer Research, National Health Research Institutes, Tainan 70456, Taiwan; bryanchen@nhri.edu.tw (S.-H.C.); kwang2@nhri.edu.tw (K.-Y.C.); 2 Department of Oncology, National Cheng Kung University Hospital, College of Medicine, National Cheng Kung University, Tainan 70456, Taiwan; 3 Division of Hematology-Oncology, Department of Internal Medicine, Chi Mei Medical Center, Liouying, Tainan 736402, Taiwan; seedvirt@gmail.com; 4 Institute of Clinical Medicine, College of Medicine, National Cheng Kung University, Tainan 70101, Taiwan; 5 Institute of Biotechnology and Pharmaceutical Research, National Health Research Institutes, Miaoli 35053, Taiwan

**Keywords:** oral squamous cell carcinoma, microbiota, lymphangiogenesis, microRNA, mitochondrion, IL-1β, tumor microenvironment

## Abstract

Oral squamous cell carcinoma (SCC) is a prevalent malignant disease worldwide, especially so in Taiwan. Early- or even preclinical-stage detection is critical for reducing morbidity and mortality from oral SCC. Epidemiological and genome association studies are useful for identifying clinicopathological risk factors for preventive, diagnostic, and therapeutic approaches of oral SCC. For advanced oral SCC, effective treatments are critical to prolonging survival and enhancing quality of life. As oral SCC is characteristic of regional invasion with lymph node metastases, understanding the aggressive features of oral SCC, particularly in lymphangiogenesis, is essential for determining effective treatments. Emerging evidence has demonstrated that the tumor microenvironment (TME) plays a pivotal role in tumor growth, invasion, and metastases. Recent clinical successes in immune checkpoint inhibitors either alone or combined with chemotherapy have also supported the therapeutic value of immunotherapy in oral SCC. This review summarizes critical advances in basic knowledge of oral SCC from the perspective of clinicopathological risk factors, molecular tumorigenesis, and the TME. We also highlight our recent investigations on the microbiome, genome association studies, lymphangiogenesis, and immunomodulation in oral SCC. This review may provide new insights for oral SCC treatment by systematically interpreting emerging evidence from various preclinical and clinical studies.

## 1. Introduction

Oral cancer is a group of malignant diseases arising from the surface of the lips, gums, tongue, mouth, and palate. As keratinocytes are the major components of the epithelium over the oral cavity, squamous cell carcinomas (SCCs) account for 90–95% of patients with this subtype of head and neck malignant diseases in histology, followed by basal cell carcinomas, mesenchymal malignancies, hematologic tumors, and melanomas [1]. Oral SCC is a perennial major public health concern because of its high prevalence worldwide. According to 2018 statistics from the International Agency for Research on Cancer [2], approximately 350,000 cases of oral cancer are newly diagnosed each year, accounting for a cumulative incidence of 4.0 per 100,000 persons. In Taiwan, betel nut consumption has led to an incidence rate of 32.46 per 100,000 persons—the highest globally [3]. Therefore, several measures, including a population-based screening program, have been employed to prevent and control oral cancers in Taiwan [4]. 

Biologically, oral cancers may develop from premalignant dysplastic lesions, which are clinically present in erythroplakia, leukoplakia, lichen planus, or combinations of these conditions [5,6]. In such cases with premalignant lesions, frequent exposure to well-established carcinogens such as alcohol, tobacco, betel nut, and human papillomavirus (HPV) infection may promote oral cancer formation. After oral SCCs are generated, tumor cells can deeply invade the local structures and lymph nodes of the neck, leading to further distant metastases. Additionally, oral cancers are characterized by “field cancerization”, meaning the aerodigestive tract of the patient is susceptible to premalignant or malignant lesions [7]. These adverse biological features strengthen the recurrence propensity of oral SCC. Although aggressive treatment strategies are employed to eradicate tumors, patients with oral cancers remain at high risk of disease recurrence [1,5,6]. Therefore, oral SCC poses a serious threat to public health, causing approximately 200,000 and 3000 annual deaths globally and in Taiwan, respectively [4,5,6]. 

**Figure 1:**
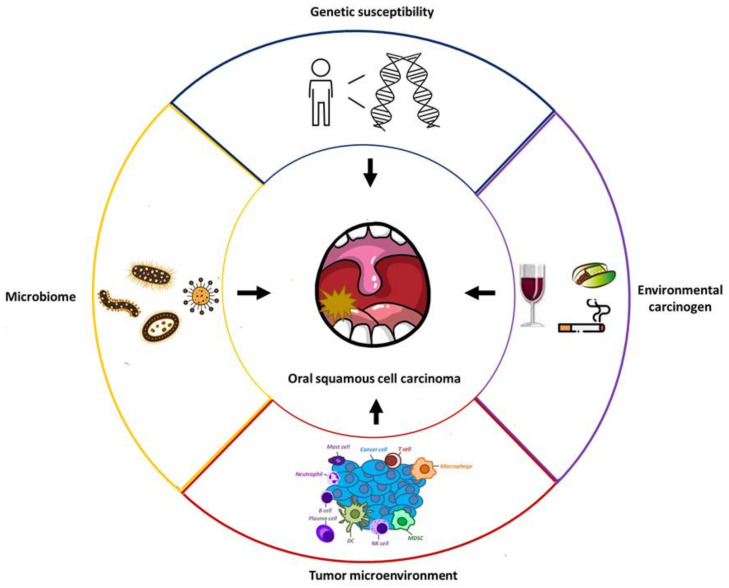
Major factors involving the formation of oral cancer. New insights into preventive, diagnostic and therapeutic strategies for oral cancer may be derived through integrative investigations on these factors.

Oral SCC treatment is dependent on disease stage, the potential complications of each therapy, and the patient’s quality of life. As early detection is the most critical step in the management of patients with cancer, an effective screening program for oral SCC is imperative [8]. To maximize the benefit of a screening program and minimize wasting of health care resources, the identification of high-risk individuals is crucial. For patients with early-stage oral SCC, surgery and radiotherapy are two principal treatment modalities for tumor eradication. As advanced oral cancers have high potential for lymph node metastases, neck dissection is a critical part of tumor surgery [9]. The level of neck dissection is determined by the size, number, and site of the lymph nodes involved. Systemic treatments, including chemotherapy, targeted therapy, and immunotherapy, are the mainstay care modalities for patients with metastatic or refractory oral SCC [10]. Some novel therapies, such as antiepidermal growth factor receptor (EGFR) or programmed cell death 1 (PD-1) antibodies, can be clinically applied; however, patients with late-stage oral cancer have limited treatment options. Therefore, the exploration of effective systemic treatments through a more comprehensive understanding of the molecular pathogenesis of oral SCC, especially the interaction between tumor cells and their microenvironment, is an unmet need. Consistent with our research interests, this review focuses on literature regarding clinicopathological risk factors, molecular tumorigenesis inside a tumor cell, and the impact of the tumor microenvironment (TME). Major factors involving the formation of oral SCC are schemed in Figure 1.

## 2. Perspectives from Clinicopathological Risk Factors

Several clinical risk factors, such as alcohol, tobacco, and betel nut use, are established in the development of oral SCC. The risk stratification of surveyed populations may enhance the effectiveness of a screening program for oral cancer. In Taiwan, a nationwide screening program for oral cancer has been operational since 2004 [4]. This program reduces the relative death risk of 47% in those with habitual betel nut use, cigarette use, or both, compared with the expected risk in the absence of screening. Epidemiological studies investigating possible causative agents are therefore essential for the early detection of cancer. In the following discussion, we highlight recent advances in microbiota and gene polymorphism in the risk estimation of oral cancer.

### 2.1. Conventional Overview of Clinicopathological Risk Factors

Tobacco exposure, betel nut use, alcohol use, and HPV infection are believed to be the main factors in the carcinogenesis of oral SCC. Among these factors, tobacco use is the greatest carcinogen, which contributes to tumor growth in various cancers [11]. Tobacco smoke is a complex mixture of over 7000 toxic chemicals, 69 of which are known to induce critical gene mutations in tumorigenesis [12]. Combined with the relevant risks of cardiovascular and respiratory diseases, the cessation of tobacco smoking is a major global public health concern. In some regions of southeast Asia, betel nut chewing with different ingredients is a common habit associated with the development of premalignant and malignant lesions in the oral cavity [8]. As is the case with tobacco, considerable evidence has indicated that the chemical components of betel nut, such as arecoline and arecaidine, can cause DNA damage that leads to oral SCC formation [13,14]. Our previous study demonstrated that nicotine-derived nitrosamine ketone and arecoline, two carcinogens related to tobacco and betel quid, can induce oral malignant transformation in animals [15]. These carcinogens can increase interleukin (IL)-1β expression in the oral mucosa cells of mice, leading to increased cellular proliferation, oncogenic cytokine stimulation, and malignant transformation. In addition to oral cancer, alcohol is a recognized carcinogen associated with several types of human cancer [16]. Alcohol use combined with tobacco use reportedly act synergistically in the increased incidence of oral cancer [8]. The major metabolite of alcohol, acetaldehyde, is mainly transformed by the enzyme alcohol dehydrogenase and then oxidized to acetate by aldehyde dehydrogenase [17]. Acetaldehyde is a genotoxic substance that can cause DNA damage in mammalian cells.

Several types of viruses have been identified as carcinogens in humans [18]. As viral proliferation in the host cell requires the breaking of both the viral and the host DNA, this integration process can cause a certain degree of DNA damage. Furthermore, virus replication can produce some oncogenic proteins that obstruct cell growth regulation. HPVs are epitheliotropic DNA viruses, particularly for keratinocytes, and are involved in the carcinogenesis of oral SCC [19,20]. In addition to oral cancer, these viruses can cause benign proliferative lesions such as papillomas, verruca vulgaris, condyloma acuminatum, and focal epithelial hyperplasia over diverse human tissues. Certain HPV serotypes, including HPVs 16, 18, 31, 33, 35, and 39, are associated with the development of premalignant and malignant lesions over the oral cavity. Among these serotypes, HPV 16 is the most commonly observed in oral cancer. HPVs are involved in cancer development through the capability of their genes and associated products to interfere with cell cycle control. HPV encodes two major oncoproteins, E6 and E7, which can impede the function of p53 and Rb tumor suppressor genes, respectively, thereby hindering cell cycle regulation. Although primary prevention with HPV vaccination has the potential to reduce the incidence of oral cancer [21], the effect of HPV vaccination on oral cancer prevention has yet to be established in major clinical trials.

### 2.2. Oral Hygiene and Microbiota

Mounting evidence has revealed that poor oral hygiene, including infrequent tooth brushing, infrequent dentist visits, and missing teeth, is associated with oral cancer [22,23,24]. Poor oral hygiene may interfere with the homeostasis of resident microbiota and induce chronic inflammation (periodontitis) in the oral environment. Inflammatory cytokines or chemokines produced in this process enable cell proliferation, oncogene activation, and tumor angiogenesis [25,26]. These microorganisms in microbial imbalance (dysbiosis) can produce carcinogens, promote carcinogenesis by other carcinogens (e.g., nitrosamines), or metabolize alcohol to genotoxic substances (e.g., acetaldehyde), subsequently leading to DNA damage. Reactive oxygen species (ROS), reactive nitrogen species, volatile sulfur compounds, and organic acids are principal carcinogens produced by oral microorganisms. Furthermore, some intracellular bacteria can promote carcinogenesis by directly controlling cell cycle regulation, apoptotic pathways and invasion ability. Of the more than 600 bacterial species constituting the oral microbiota, *Fusobacterium nucleatum*, *Porphyromonas gingivalis*, and *Prevotella intermedia* are the most represented bacteria types associated with oral SCC formation [26]. In our recent report investigating saliva samples from patients with cancer and healthy volunteers, we discovered that three periodontopathogenic bacteria species (*Prevotella tannerae*, *F. nucleatum*, and *P. intermedia*) were correlated with an increased risk of oral SCC. A patient with percentages of the three species all above the median had a 2.3 times higher oral SCC risk (odds ratio = 2.34; 95% confidence interval: 1.28–4.26) [27]. Moreover, harmful lifestyle factors, including poor oral hygiene and cigarette, alcohol, and betel nut use, were associated with the presence of these three bacteria species in people’s saliva. Notably, the percentages of periodontopathogenic bacteria were positively correlated with the expression levels of IL-1β in the examined saliva (Pearson coefficient = 0.42; *p* = 0.0009). These results support the connection among risky life habits, microbial dysbiosis, and IL-1β stimulation in the constitution of a TME conducive to oral SCC. The bacterial species reportedly relevant to oral SCC are summarized in Table 1 [27,28,29,30,31,32,33].

### 2.3. Gene Susceptibility

Genomic variation among individuals may induce distinct physiological responses to environmental stimuli. Genetic variations, such as single nucleotide polymorphism (SNP), may influence the transcription efficiency of genes as well as the functions and quality of the resulting proteins. Therefore, genetic variations may lead to disparate disease susceptibility among individuals. As the carcinogenic mechanism of tobacco, alcohol, and betel nut is mainly through DNA damage, defects in the DNA damage repair network are associated with genotoxic susceptibility to oral cancer. XRCC1 and XRCC3 participate in the base excision repair system for the repair of DNA single strand break, and the SNPs of these genes are associated with the risk of oral SCC [34,35]. Moreover, several metabolite enzymes are responsible for neutralizing and eliminating toxic chemicals in the human body. These enzymes are typically classified into phase I (activation) and phase II (neutralization) enzymes [36]. The correlation between the risk of oral cancer and the SNPs of these metabolic enzymes is commonly investigated. Phase I enzymes are principally involved in increasing the water solubility of lipophilic xenobiotic enzymes and providing sites for conjugation reactions to phase II enzymes. Members of the cytochrome P450 family are phase I enzymes, and genetic polymorphisms of these genes are commonly studied in the carcinogenesis of various cancers. CYP1A1 is a member of the P450 family, the genetic variations of which are associated with the pathogenesis of oral cancer [37]. Phase II enzymes include members of various transferases responsible for the elimination of toxic chemicals. Among these phase II enzymes, the SNPs of *GSTT1* and *GSTM1* are well investigated in the initiation of oral SCC [37,38,39]. Details of these SNPs are summarized in Table 2.

Toll-like receptors (TLRs) play a critical role in the human immune system against microbial infection and wound healing process in tissue injury [40,41]. The overexpression of several TLRs is positively correlated with oral SCC risk [42]. Some evidence has indicated that oral bacteria interact with the TLRs and promote inflammation of the oral epithelium [43]. Our previous report also demonstrated the interaction between TLR polymorphisms and the oral SCC risk associated with oral bacteria [27]. Individuals with the combined SNPs of *TLR2* and *TLR4* had an elevated risk of bacteria-related oral cancer (odds ratio = 1.46; 95% confidence interval: 1.23–1.75). These results provided biological evidence to connect poor oral hygiene, gene susceptibility, and oral SCC risk. For brevity, other major genetic variations relevant to oral SCC are presented in Table 2 [44,45,46].

## 3. Perspectives on Molecular Tumorigenesis

Carcinogenesis is a complex biological process in which some genetic or epigenetic events alter the regulation of sustaining proliferate signaling, evading growth suppressors, resisting apoptosis, enabling replicative immortality, promoting genomic instability, reprogramming energy metabolism, inducing angiogenesis, activating invasion capacity, tumor-promoting inflammation, and escaping immune surveillance [47]. The malignant transformation of oral SCCs is also the cumulative result of dysfunction in these critical biological responses to the stimuli from endogenous or exogenous carcinogens. Comprehensive understanding of these molecular features in oral SCCs not only provides information on their malignant behaviors but also suggests the key elements of potential therapies. 

### 3.1. General Concepts of Molecular Tumorigenesis Inside Oral Cancer Cells

Sustained proliferation of tumor cells is the fundamental characteristic of cancer formation, and enhanced mitogenic signaling is the central element of neoplastic cell growth. Cancer cells can amplify growth signals by increasing growth factor ligand production, overexpressing growth receptors on the cell surface, modifying the structure affinity of the receptor, or intensifying intracellular signaling transductors [47]. Several aberrations of oncoproteins or proto-oncoproteins, such as EGFR, K-ras, c-myc, FGF3, and cyclin D1, have been identified in the development of oral SCC [48,49,50]. Among these aberrations, the amplification of cellular EGFR is particularly critical in clinical applications. EGFR overexpression can be observed in 80–90% of patients with head and neck cancer (HNC) and associated with poor overall survival in clinical practice [51,52]. Currently, an anti-EGFR antibody, cetuximab, is approved for the treatment of patients with advanced HNC. In a landmark phase III clinical trial that recruited 442 patients with recurrent or metastatic HNC, the addition of cetuximab to platinum-based chemotherapy significantly extended the overall survival of patients in the experimental group (hazard ratio: 0.80; 95% confidence interval: 0.64–0.99; *p* = 0.04) [53]. Oral cancer accounted for approximately 20% of the study population in that clinical trial, and subgroup analyses revealed that the combination therapy of cetuximab and chemotherapy had the greatest impact on survival extension in patients with oral cancer (hazard ratio: 0.42; 95% confidence interval: 0.26–0.67). Therefore, the anti-EGFR antibody is currently a key systemic therapy for patients with advanced oral SCC [54]. 

To maintain tumor growth, neoplastic cells must elude the robust cell cycle checkpoint regulation process. This process is strictly controlled by the genetic products of tumor suppressor genes. The inactivation of p53, which can result in sustained cell proliferation and inhibition of apoptosis signaling, is the most common genetic alteration observed in all human cancers [55]. Dysregulation of the *p53* gene, including point mutation or deletion, can be observed in more than 50% of oral cancer tissues [56]. After p53, *CDKN2A* is the second most frequently mutated gene in oral SCC [57]. During the G1 to S phase of the cell cycle, the product encoded by *CDKN2A*, namely p16, can disrupt the interaction between CDK4/6 and cyclin D1 and subsequently promote cell cycle progression. In a study examining tumor tissues, the loss of *CDKN2A* function, which is regulated by gene mutations, hemizygous or homozygous loss, and promoter methylation, can be distinguished in approximately 90% of oral SCCs [58]. A recent phase II clinical trial demonstrated the potential application of the cell cycle inhibitor palbociclib in the treatment of oral cancer [59]. In this clinical study, 62 patients with refractory HPV-unrelated HNC were enrolled, including 26 patients with oral cancers. The combination of cetuximab and palbociclib, a CDK4/6 inhibitor, achieved significant responses of 39% and 19% in patients with platinum- and cetuximab-resistant HNC, respectively. These results underscore the biological significance of tumor suppressor genes, especially cell cycle regulators, in the malignant transformation of oral SCC.

### 3.2. Molecular Overview of Lymph Node Metastases of Oral Cancer

Lymphovascular invasion is a crucial step in the metastasis of human cancers. Due to the abundance of the lymphatic vessels in the cervix, lymph node metastases frequently occur in patients with HNC and determine their prognosis and therapeutic strategies [60,61,62]. A comprehensive understanding of the molecular mechanisms of lymphovascular invasion is crucial in the care of patients with oral cancer. From the perspective of cancer hallmarks, the induction of angiogenesis and invasion activity is the major determinant in tumor lymphovascular invasion. Among all proangiogenic factors, vascular endothelial growth factor C (VEGF-C) is a critical lymphangiogenic inducer in human cancers, including oral SCC [63,64,65]. Through the activation of vascular endothelial growth factor receptor 3 on the lymphatic endothelium, neoplastic cells can express VEGF-C to induce the growth and migration of lymphatic vessels. In a clinical correlation study, VEGF-C overexpression was an independent prognostic factor for poor survival in patients with oral SCC [64,65]. Some proangiogenic factors have been reported in the development of lymphangiogenesis in oral cancer, such as prospero homeobox 1, forkhead box C2, and astrocyte elevated gene-1 [66,67]. These factors induce the lymphangiogenesis of oral SCC cells by activating VEGF-C expression. In addition to the angiogenic switch, epithelial–mesenchymal transition (EMT) is a well-known dynamic process enabling cancer cells to undergo multiple biochemical changes that lead to enhanced migratory ability, invasive capacity, and extracellular matrix component production [68]. In the EMT stage, epithelial cancer cells can shift to mesenchymal phenotypes through, for example, the loss of their intercellular junctions and migratory polarity. Through the EMT process, cancer cells can invade lymphovascular circulation and migrate to local lymph nodes or result in distant organ metastases. Among several EMT-associated transcription factors, the activation of Snail is the most important mediator of the enhanced invasiveness of oral cancer. Related reports have demonstrated that Snail overexpression can promote stemness phenotypes and resistance to various types of therapies in oral SCC cells [69,70]. Two members of the Twist family, Twist 1 and Twist 2, are also correlated with mesenchymal phenotypes of oral SCC [71]. Clinical studies have revealed that Twist overexpression is an unfavorable factor for survival outcomes in patients with oral cancer [72,73]. 

### 3.3. Novel Investigations of Lymphovascular Invasion in Oral Cancer

Several reports have indicated the major role of the Wnt pathway in regulating the invasiveness and stemness phenotypes of oral cancer [74,75]. This signaling pathway is generally divided into two subtypes: β-catenin-dependent signaling (or canonical Wnt signaling) and β-catenin-independent signaling (or noncanonical Wnt signaling). In the canonical pathway, the Wnt family proteins first bind to their receptors on the cell membrane; they subsequently induce the β-catenin signalizing in the nucleus and finally activate the T-cell/lymphoid-enhancing-factor transcription factors. For noncanonical Wnt signaling, the Wnt proteins activate downstream signaling by binding to Frizzled receptors and other transmembrane coreceptors, such as Ror1, Ror2, RYK, and low-density lipoprotein receptor-related protein 5/6. A total of 19 Wnt family proteins have been identified as initiating this signaling pathway [76]. In our previous report, we observed that the high levels of Wnt5B protein in serum were associated with lymph node metastasis in Taiwanese patients with oral SCC [77]. Moreover, Wnt5B can induce lymphangiogenesis and EMT phenotypes in oral cancer by regulating the expression of Snail and Slug proteins. These results support the involvement of the Wnt pathway in promoting lymph node metastases of oral cancer. To further understand the underlying mechanisms of lymphovascular invasion, we previously developed LN1-1 cells possessing lymphangiogenesis and lymphatic metastasis potential from OEC-M1 cells [78]. By using a liquid chromatography–tandem mass spectrometry (LC-MS/MS)-based proteomic platform, we identified high expression levels of interferon-stimulated gene 15 (ISG15) in LN1-1 cells [78]. In cellular biology, ISG15 acts as a ubiquitin-like protein regulated by interferon (IFN) expression [79]. In our clinical analyses of the oral SCC tissues of Taiwanese patients, we noted increased ISG15 expression, which was associated with poor survival outcomes [78]. In mechanistic studies, ISG15 was found to contribute to lymphovascular invasion by regulating Rac1 activity in oral SCC cells. Rac1 protein, which belongs to the Rho family of GTPases, is involved in the tumorigenic and migratory phenotypes of cancer cells [80]. Our results revealed that ISG15 is a potential biomarker for detecting lymph node metastases and predicting prognoses of oral cancer. Notably, recent studies have demonstrated that extracellular vesicles (EVs) are essential mediators in communication between cancer cells and their neighboring cells in the TME [81,82]. EVs are lipid-bilayered vesicles 30–150 nm in diameter that are filled with bioactive molecules, including proteins, lipids, and nucleic acids. EVs are referred to as “oncosomes” if the molecules they contain are involved in the malignant transformation of cancer cells. We previously discovered that LN1-1 cell-derived EVs can promote the migration and tube formation of lymphatic endothelial cells [83]. We identified high levels of laminin γ2 by using a mass spectrometry-based proteomic platform in EVs from LN1-1 cells and plasma of Taiwanese patients with lymph node metastasis. The suppression and inhibition of laminin γ2 were found to impair LN1-1 EV-mediated lymphangiogenesis in oral SCC. These results highlight the importance of oncogenic EVs in regulating lymph node metastases of oral cancer. The biomolecules that may be associated with the lymphovascular invasion of oral SCC are summarized in Table 3.

MicroRNAs (miRNAs) are a family of small noncoding RNAs that are encoded by endogenous genes and involved in several biological processes, including carcinogenesis [84,85]. These noncoding RNAs, generally composed of 18–22 nucleotides, can suppress the expression of corresponding genes by targeting the 3′-untranslated regions of mRNA and subsequently regulate the malignant behaviors of cancer cells. In oral cancer, several oncogenic or tumor-suppressive miRNAs have been reported, and investigations on how these small RNAs regulate lymph node metastasis have been noteworthy [86,87,88]. To explore the biological function of miRNA in regulating lymph node metastasis in oral cancer, we analyzed the gene expression profiles of a Taiwanese cohort that were established using genomic DNA and mRNA from 40 tumor tissues and matched oral mucosa [89,90,91,92]. Through a series of comprehensive investigations integrating bioinformation analyses, clinical specimen studies, in vitro function assays, and in vivo animal experiments, several miRNAs have been identified to be involved in the lymph node metastasis of oral cancer. Relevant to the crucial mediating role of Wnt pathways in lymphangiogenesis, our previous report revealed that miR329 and miR410 can modulate the invasion capacity of oral SCC cells by targeting Wnt7B, an inducer of the Wnt family [89]. In the aforementioned study, arecoline, a major alkaloid of betel nut, was observed to reduce miR329 and miR410 expression levels, which can thus result in the enhanced invasion activity of oral SCC cells. Increasing evidence has also revealed that transforming growth factor-β (TGF-β), a crucial cytokine regulating inflammatory responses in the TME of various human cancers, can influence the tumorigenesis of oral cancer [93,94]. Upon activation, ligands of the TGF-β family interact with receptor types I and II and begin signaling transduction in the SMAD (canonical) or non-SMAD pathway. In the canonical pathway, the members of the SMAD family, including SMAD2, SMAD3, and SMAD4, interact and translocate into the nucleus to control gene expression. Through interaction with other transcription factors, the TGF-β pathway can regulate the expression levels of genes participating in proliferation, apoptosis, invasion, and immune responses. Our previous report demonstrated that higher miR-455-5p expression is correlated with lymph node metastases in Taiwanese patients with oral cancer [90]. Mechanistic studies have revealed that miR-455-5p is regulated by SMAD3 expression and involved in the TGF-β pathway of oral SCC cells. Moreover, a Runt family transcription factor, RUNX2, is reportedly a central modulator correlated with numerous physiological functions, such as osteoblast differentiation, chondrocyte maturation, cytoskeleton remodeling, and cellular movement [95]. Our previous study indicated that downregulated miR-376c can promote lymph node metastasis in oral SCC by targeting the RUNX2/Activin-A axis [91]. Similar to TGF-β, tumor necrosis factor-α (TNF-α) is a crucial inflammatory cytokine involved in malignant transformation of human cancers [96,97]. By activating the mitogen-activated protein kinases (MAPK) pathway or other signaling to induce EMT, TNF-α promotes the invasive and metastasis capacity of cancer cells. Our recent report revealed that an oncogenic miRNA, miR-450a, is involved in TNF-α pathway-mediated motility of oral SCC cells [92]. Transmembrane proteins (TMEMs) are a group of proteins spanning the lipid bilayer and correlating with cell differentiation and tumorigenesis [98,99,100]. In oral SCC cells, TNF-α induces miR-450a expression to enhance invasion ability by abolishing TMEM182 expression. The major miRNAs involved in lymph node metastases of oral cancer are summarized in Table 4.

## 4. Overview of the Impact of the TME on Oral SCC

Mounting evidence has indicated that tumor growth relies on not only dysregulated biological responses inside a cancer cell but also the impact from the intricate ecosystem in the TME [101,102]. To avoid immune effector cell attack, cancer cells must create an immunosuppressive milieu through the complex interplay between tumor cells and surrounding cells in the TME, which includes loss of tumor neoantigen, polarization of immune cells, dysregulation of inflammatory cytokines, and induction of immune checkpoints. Here, we summarize major cell components and cytokines contributing to the immunosuppressive TME of oral cancer.

### 4.1. Current Achievements and Limitations of Immunotherapy

Cytotoxic T cells are a major cell type responsible for immunological antitumor responses [103,104]. Immune checkpoints on cytotoxic T cells, such as PD-1, CTLA-4, TIM-3, LAG-3, TIGIT, GITR, and VISTA, are immunosuppressive molecules that help to maintain host tolerance by attenuating T-cell function. However, cancer cells can leverage this biological feature to escape attack from immune cells. In patients with oral cancer, emerging evidence has demonstrated that immune checkpoints are the critical mechanism leading to cancer cell evasion from immunosurveillance [103,104]. Among various immune checkpoints, PD-1 and its ligand PD-L1 have attracted substantial research interest because blocking this immunosuppressive pathway has strong antitumor effects. Some immune checkpoint inhibitors (ICIs) targeting the PD-1/PD-L1 axis have been developed for treating several human cancers [105,106]. In patients with HNC, the clinical benefits of anti-PD-1 antibodies, such as nivolumab and pembrolizumab, have been demonstrated in several reports [107,108,109]. The results of the CheckMate 141 and KEYNOTE-040 trials have indicated that both nivolumab and pembrolizumab treatment can prolong overall survival in patients with HNC refractory to a platinum-based regimen compared with a control group (hazard ratio for nivolumab: 0.70; *p* = 0.01; hazard ratio for pembrolizumab: 0.80; *p* = 0.0161) [107,108]. The KEYNOTE-048 study, in which patients with oral cancer comprised approximately 30% of all participants, has changed the paradigm regarding first-line therapy in recurrent or metastatic HNC. The final results of this landmark study indicated that compared with cetuximab combined with chemotherapy, the combination treatment with pembrolizumab and chemotherapy led to superior overall survival in those with a PD-L1 combined positive score (CPS) ≥ 20 (hazard ratio: 0.60; *p* = 0.0004) and CPS ≥ 1 (hazard ratio: 0.65; *p* < 0.0001) as well as in the total population (hazard ratio: 0.77; *p* = 0.0034) [109]. On the basis of the results of these clinical studies, anti-PD-1 antibodies have been indicated in patients with advanced HNC. However, room remains for improving ICI treatment in patients with oral cancer because a response rate of only approximately 20% can be achieved in clinical practice [110]. Several action mechanisms, such as the recruitment of immunosuppressive cells and increased expression of immunoinhibitory signals, have been suggested to contribute to tumor resistance to ICI treatment [111]. A comprehensive understanding of the complex immune network in the TME is essential for improving immunotherapy efficacy in patients with oral cancer. Recent advances of anticancer drugs for patients with advanced HNC are summarized in Table 5.

### 4.2. Outlook on the Immunosuppressive Network in the TME 

In addition to cancer cells, the stroma of the TME is composed of several cellular components, including cancer-associated fibroblasts (CAFs), effector or regulatory T cells, M1/M2 macrophages, myeloid-derived suppressor cells (MDSCs), N1/N2 neutrophils, natural killer cells, and mast cells [101,102]. These diverse types of cells can interact through the complex connection network consisting of growth factors, chemokines, cytokines, and proteins of the extracellular matrix. In general, effector T cells, M1 macrophages, N1 neutrophils, and natural killer cells have antitumor growth functions, whereas CAFs, regulatory T cells, M2 macrophages, MDSCs, N2 neutrophils, and mast cells are tumorigenic. An immunosuppressive TME is thus the result of the intricate interaction between numerous immunogenic types of cells and molecules. A recent gene profiling study that recruited 1380 patients identified two immune molecular subtypes in HNC: those characterized by active or exhausted immune responses [112]. An analysis of data from RNA sequencing of these tumors revealed that those with Active Immune Class, which are characterized by enriching proinflammatory M1 macrophage signatures, abundant tumor infiltrating lymphocytes, and a high proportion of HPV infection, had a favorable prognosis and potential response to ICI treatment. By contrast, Exhausted Immune Class through enrichment of the inflammatory M2 macrophage signature as well as Wnt/TGF-β signaling pathway activation were associated with poor survival outcomes. Therefore, precisely determining the unique immunogenic background of each patient is crucial for the application of immunotherapies in oral cancer.

The immune network constructed by multiple cytokines can regulate cellular biological responses and contribute to an immunosuppressive TME. Among several immunomodulatory cytokines, TGF-β is particularly critical in regulating tumor immunity. Notably, TGF-β acts as a tumor suppressor during early-stage oncogenesis in normal epithelial cells [93,94]. However, in late-stage oncogenesis, tumor cells lose their growth inhibitory response to TGF-β. In addition to EMT promotion, TGF-β regulates several immune cell functions in the TME of oral cancer by, for example, hampering antigen presentation by dendritic cells, inducing M2 macrophages, and facilitating the differentiation of regulatory T cells [94,101,102]. Several reports have indicated that IL-6, derived from CAFs, is involved in the shaping of the immunosuppressive TME of oral cancer [113,114]. By activating JAK/STAT3 signaling, IL-6 promotes tumorigenesis and negatively regulates the function of neutrophils, natural killer cells, and effector T cells [115]. IL-8 is a proangiogenic and proinflammatory mediator during the angiogenesis induction of oral SCC [116,117]. By activating the MAPK pathway, oral SCC cells can use IL-8 to induce angiogenic activity in M2 macrophages. In oral cancer, IL-10 is also crucial in promoting the polarization of M2 macrophages and impairing the cytotoxic function of effector T cells [102,118]. Clinical studies have reported that high IL-10 expression is correlated with poor outcomes in HPV-unrelated oral SCC, especially for patients in whom INF-γ secretion and TGF-β levels are low [119,120].

### 4.3. Role of IL-1β in Immunomodulation of Oral Cancer

**Figure 2:**
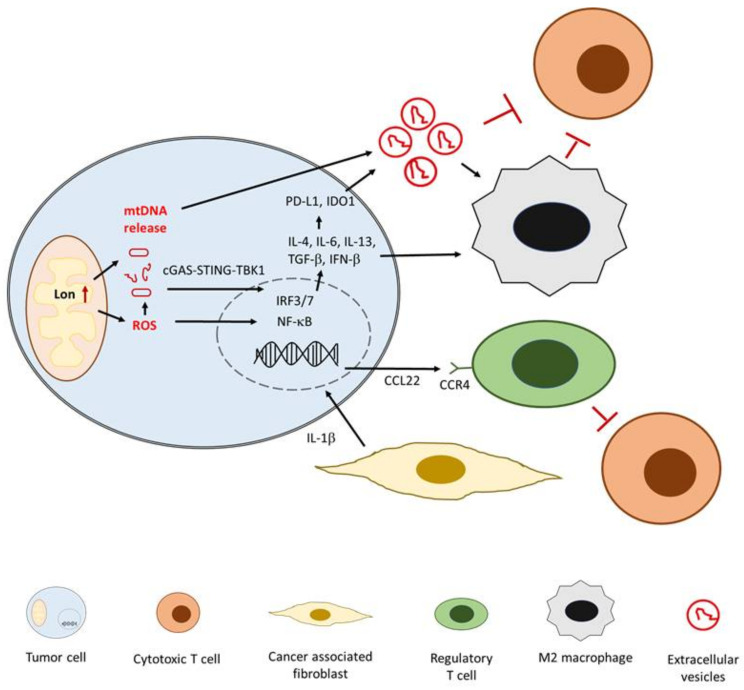
Schematic representation of IL-1β and mitochondrial Lon’s regulation of an immunosuppressive TME. Red arrow indicates overexpression; black arrow indicates activation or promotion. Abbreviations: CCL22 = C-C motif chemokine ligand 22, CCR4 = C-C chemokine receptor type 4, IRF3/7 = IFN regulatory factor-3 and 7, ROS = reactive oxygen species, mtDNA = mitochondrial DNA.

Our previous studies have revealed that IL-1β plays a major role in oral cancer tumorigenesis [15,27]. Increased IL-1β expression was observed in a mouse oral SCC model treated with 4-NQO and arecoline and was associated with the severity of oral malignant transformation [15]. IL-1β levels are also correlated with the percentages of periodontopathogenic bacteria in the saliva of patients with oral cancer; moreover, IL-1β stimulation regulates multiple immunogenic responses in the TME, such as enhancing IL-6 and IL-8 expression, promoting M2 macrophage polarization, and inducing Myc-dependent angiogenesis [15,121,122]. Through IL-1β reaction, CAFs induce NF-κB-mediated C-C motif chemokine ligand 22 expression and further promote immunosuppressive activity of regulatory T cells [123]. A clinical study using a proinflammatory panel of cytokines for evaluation indicated that IL-1β expression serves as a prognostic marker for patients with HNC [124]. The role of IL-1β in regulating immunosuppressive TME and promoting tumor progression is illustrated in Figure 2.

### 4.4. Metabolism Reprogramming and Antitumor Immunity in Oral Cancer

During tumor progression, cancer cells experience hypoxia and environmental stress, resulting in a lack of energy for cell growth and proliferation [47,125,126]. The metabolic reprogramming of cancer cells has been implicated as a crucial mechanism of cell survival under environmental stress. The Warburg effect, defined as increased glucose metabolism and lactate production for ATP generation by cancer cells, is a significant biological feature of metabolic reprogramming. Accumulating evidence suggests that in addition to proliferation and survival, cancer metabolism is correlated with antitumor immunity in the TME. Through the release of signaling molecules and the expression of immune ligands by metabolism reprogramming, an immunosuppressive TME can be generated during tumor progression. In a study examining oral SCC metabolism, CAF-secreted hepatocyte growth factor increased lactate levels in the TME and facilitated tumor progression as a result of increasing glycolysis of cancer cells [127]. Notably, through the induction of fibroblast growth factor, CAFs are more efficient in using lactate as a carbon source than are oral SCC cells. In addition to the effect on CAFs, lactate accumulation in the extracellular medium modifies monocyte function. Increased levels of extracellular lactate can reportedly interfere with macrophage polarization to the immunosuppressive M2 subtype [128]. Furthermore, mitochondria are major organelles involved in cellular metabolism. Recent studies have revealed that the mitochondrial metabolism of macrophages in response to various levels of stress can drive M1/M2 polarization in the TME [129,130]. The mitochondria are also the major origin for the production of endogenous ROS, which have recently been reported to be involved in the formation of an immunosuppressive TME [131,132,133]. The ROS production of cancer cells can impair effector T-cell function through the induction of PD-1 expression and immunosuppressive activity of regulatory T cells. 

Inside the mitochondria, the protease Lon is a crucial regulator responsible for mitochondrial metabolism and hemostasis [134,135]. Owing to its chaperone activity, mitochondrial Lon controls mitochondrial protein quality, mitochondrial DNA (mtDNA) repair, and ROS production. Therefore, several studies have attempted to elucidate the critical role of mitochondrial Lon in tumorigenic activity, focusing on proliferation, antiapoptosis, and EMT [134,135]. Recent studies have revealed a novel connection between mitochondrial Lon and an immunosuppressive TME in oral cancer [136,137]. In our previous study, mitochondrial Lon induced the ROS-mediated production of several inflammatory cytokines, including TGF-β, IL-6, IL-13, and VEGF-A, which further promoted EMT, angiogenesis, and M2 macrophage polarization in oral SCC cells [136]. Moreover, M2 macrophages induced Lon expression and subsequently activated themselves, leading to an immunosuppressive TME. In addition to M2 macrophage polarization, mitochondrial Lon controlled antitumor immunity through mtDNA release and EV secretion. Our recent report revealed that Lon-induced mtDNA release can increase IFN signaling via the cGAS–STING–TBK1 pathway in oral SCC cells [137]. The activation of IFN signaling upregulates PD-L1 and IDO-1 expression, thus inhibiting effector T-cell activity. Mitochondrial Lon also induces EVs containing mtDNA and PD-L1 proteins, and activates M2 macrophages to inhibit T-cell function through the secretion of IFN and IL-6. The interaction between mitochondrial Lon and immunosuppressive TME is elucidated in Figure 2.

## 5. Future Perspectives

Early detection and effective treatment remain major goals in the management of patients with oral SCC. With advances in knowledge regarding molecular tumorigenesis, research on oral SCC increasingly targets the cumulative effects of multiple influential factors coexisting in the TME instead of single tumor cell. Therefore, the identification of risky genetic variants, somatic genomic alterations, dysregulated signaling pathways, pathogenic microbiomes, and the characterization of key components in the TME are of utmost importance to support relevant advances in clinical management of oral SCC. In fact, in this era replete with “omics,” such as genomics, epigenomics, transcriptomics, proteomics, and metabolomics, large-scale datasets generated using high-throughput analytic techniques facilitate major advances in identifying potential diagnostic and therapeutic targets for human cancers. For diagnostic purposes, the analyses of nontumor biological specimens, especially blood and saliva, have attracted considerable attention as a useful tool for the early detection or monitoring of oral cancer. In these easily accessible biological specimens, various useful elements, such as circulating tumor cells, tumor genetic material, specific proteins, EVs, and microbiomes can be assessed for clinical applications. Extending clinical indication of immunotherapy in oral cancer is a promising strategy for improving survival outcomes. Accordingly, exploring the intricate immune network regulating tumor growth is a critical task in cancer management. By using a high-throughput sequencing platform, the single-cell RNA-seq (scRNA-seq) technique can be a powerful tool for analyzing genomics, epigenomics, and transcriptomics at cellular resolution [138,139]. This rapidly evolving technique can lead to innovative discoveries in tumor biology, immuno-oncology, and therapeutic resistance. In a recent study examining our previously established oral SCC mouse model, the scRNA-seq technique demonstrated robust analytic value in determining the oncogenic signaling pathway and therapy resistance [139]. Enrichment analysis of gene expression profiling revealed that the MYC signaling pathway is predominant in clusters of stem cells and keratinocytes. The gene signatures identified from oral SCC-like cells were also comparable to those of cancer cells with drug resistance. These results suggest the potential utility of the high-throughput sequencing tool in analyzing complex immune contexture and predicting therapy response in oral cancer.

Although the clinical benefits of ICI treatments have been proven, several major roadblocks, such as relatively low clinical response and acquired drug resistance, may limit their clinical utility in oral cancer [10,107,108,109]. The careful design of clinical trials on the basis of a comprehensive understanding of the immunosuppressive network in the TME is pivotal for improving the clinical efficacy of ICI treatment. As immunosuppressive cellular components or cytokines are central to the dysfunction of effector T cells, therapies targeting these immune inhibitory factors should be considered for clinical applications. Among various immunosuppressive cell types, numerous clinical studies targeting M2 macrophages and CAFs are ongoing. Given that colony stimulating factor 1 (CSF1) is required for the recruitment and differentiation of macrophages in the TME, the blockade of CSF1 receptor (CSF1R) has been surveyed for its therapeutic potential [140,141]. The clinical safety and efficacy of some anti-CSF1R antibodies or specific inhibitors, such as emactuzumab (NCT02323191) and pexidartinib (NCT04488822), are currently being investigated in phase I trials. In addition to the identification of pathogen-associated components, TLRs can regulate the polarization of macrophages to the M1 subtype with antitumor activity [141]. Indeed, a phase 1b/2 trial was conducted to evaluate a TLR9 agonist, SD-101, in combination with pembrolizumab in immunotherapy-naïve patients with advanced HNC [142]. In this early-phase trial, the combination treatment with SD-101 and pembrolizumab achieved a 48% disease control rate and satisfactory toxicity profile. The safety and immune responses of the TLR8 agonist motolimod combined with nivolumab are currently being investigated in a phase I trial of patients with HNC (NCT03906526).

In general, the functions of CAF-targeting anticancer treatments are mainly through the exhaustion of CAFs by the deletion or inhibition of cell surface markers and inactivation of CAF-associated chemokines, cytokines, or extracellular matrix (ECM) proteins [143]. As fibroblast activation protein (FAP) is a key player in ECM remodeling and CAF function, several approaches involving CAF inactivation have been developed by targeting this surface marker [144]. In a phase I trial, RO6874281, an engineered variant of interleukin-2 fused with an anti-FAP antibody produced a long-term response in one patient with HNC [145]. A phase II basket trial recruiting patients with solid tumors, including HNC, to evaluate the efficacy of combination treatment with RO6874281 and an anti-PD-L1 antibody (atezolizumab; NCT03386721) is ongoing. Additionally, accumulating evidence has demonstrated the vital role of IL-1β in mediating M2 macrophages and CAFs in the TME [121,122,123]. The role of IL-1β blockade in cancer treatments is a promising research area. Clinical trials evaluating the efficacy of anti-IL-1β antibodies, such as canakinumab (NCT02900664, NCT03447769, NCT03631199, and NCT03626545) and gevokizumab (NCT03798626), are ongoing with various treatment strategies in patients with certain types of cancer; the findings of these studies are likely to have major implications for oral cancer treatment. 

## 6. Conclusions

Oral SCC continues to be a life-threatening disease worldwide. The early detection and diagnosis of human cancers remain key to reducing cancer-associated mortality. Research has confirmed that this malignant disease arises because of exogenous carcinogen exposure (e.g., tobacco, betel nut, alcohol, and HPV), chronic inflammation induced by pathogenic microbiota, and the genetic susceptibility of the host. These genotoxic events can irreversibly damage the genetic material of keratocytes and promote malignant transformation. A comprehensive understanding of these clinicopathological risk factors can facilitate the identification and even prevention of oral SCC at the preclinical stage. Beyond the cancer cell itself, emerging evidence has indicated that the intricate network of cellular or noncellular components in the TME can determine the growth and metastasis of cancer cells. As ICI treatments have promising benefits for patients with oral SCC, exploring the mechanisms for creating an immunosuppressive TME is imperative. Further clinical trials with immunotherapy-based designs are also encouraged. With the advances in the high-throughput sequencing technique, considerable information from genomics, epigenomics, transcriptomics, metabolomics, and microbiomes can be useful for the design of novel diagnostic or therapeutic approaches to oral SCC. Therefore, determining how to incorporate emerging knowledge from epidemiology, tumor biology, genome-wide association studies, the microbiome, and immuno-oncology is a key step in effectively combating oral SCC.

## Figures and Tables

**Table 1:** Examples of bacterial species associated with oral cancer development.

Bacterial Species	Characteristic	Sample Type	Reference
*Actinomyces* spp.	Gram-positive anaerobe	Saliva	[28]
*Clostridium* spp.	Gram-positive anaerobe	Saliva	[28]
*Capnocytophaga* spp.	Gram-negative bacteria	Premalignant lesion, tumor, and saliva	[29,30,31]
*Enterobacteriaceae* spp.	Gram-negative bacteria	Saliva	[28]
*Fusobacterium nucleatum*	Gram-negative anaerobe	Saliva and oral rinse	[27,32]
*Porphyromonas gingivalis*	Gram-negative anaerobe	Saliva and tumor	[31,33]
*Prevotella intermedia*	Gram-negative anaerobe	Saliva and oral rinse	[27,32]
*Prevotella melaninogenica*	Gram-negative anaerobe	Saliva	[31]
*Prevotella tannerae*	Gram-negative anaerobe	Saliva	[27]
*Streptococcus* spp.	Gram-positive anaerobe	Premalignant lesion, tumor, and saliva	[29,30,31]
*Veillonella* spp.	Gram-negative anaerobe	Oral rinse	[32]

**Table 2:** Examples of gene polymorphisms associated with oral cancer development.

Gene Name	Polymorphism	Protein Function	Reference
*XRCC1*	Arg399Gln	DNA damage repair	[34]
*XRCC3*	Thr241Met	DNA damage repair	[35]
*CYP1A1*	MspI site	Phase I enzyme	[37]
*GSTM1*	Null genotype	Phase II enzyme	[37]
*GSTT1*	Null genotype	Phase II enzyme	[37]
*TNF-*α	−238G > A	Inflammation	[44]
*COX-2*	−765G > C, +837 T > C	Inflammation	[45]
*RETN*	A/T/G/G haplotype	Inflammation and metabolism	[46]

**Table 3:** Major biomolecules involved in lymph node metastasis in Taiwanese patients with oral squamous cell carcinoma (SCC).

Molecule Name	Biological Function	Potential Mechanisms Regulating Lymph Node Metastasis	Reference
Wnt5B	Activator of the Wnt pathway	Promotes lymphangiogenesis and endothelial–mesenchymal transition by regulating the expression of Snail and Slug proteins	[77]
ISG15	Ubiquitin-like protein	Induces lymphovascular invasion by targeting Rac1 activity	[78]
Laminin γ2	Basement membrane protein	Enhances lymphangiogenesis through the uptake of laminin γ2-enriched EVs by lymphatic endothelial cells	[83]

**Table 4:** Major miRNAs involved in lymph node metastasis in Taiwanese patients with oral SCC.

miRNA Name	Biological Function	Potential Mechanisms Regulating Lymph Node Metastasis	Reference
miR329 and miR410	Tumor suppressor microRNA	Reduced expression of miR329 and miR410 activates the Wnt pathway by targeting Wnt7B	[89]
miR-455-5p	Oncogenic microRNA	Upregulated by SMAD3 expression and involved in TGF-β pathway Regulates cellular biology by targeting UBE2B	[90]
miR-376c	Tumor suppressor microRNA	Downregulated miR-376c promotes lymph node metastasis by targeting the RUNX2/Activin-A axis	[91]
miR-450a	Oncogenic microRNA	Induced by the TNF-α pathway Enhances invasion ability by abolishing TMEM182 expression	[92]

**Table 5:** Novel anticancer drugs achieving clinical benefits in patients with advanced head and neck cancer (HNC).

Drug	Action Mechanism	Clinical Benefit	Reference
Cetuximab	Antiepidermal growth factor receptor antibody	In the EXTREME study, the addition of cetuximab to platinum-based chemotherapy in first-line therapy extended the overall survival of patients with recurrent or metastatic disease (hazard ratio: 0.80; 95% confidence interval: 0.64–0.99; *p* = 0.04).	[53]
Nivolumab	Antiprogrammed cell death protein 1 antibody	In the CheckMate 141 study, nivolumab monotherapy prolonged overall survival in patients with advanced disease refractory to a platinum-based regimen (hazard ratio: 0.70; 95% confidence interval: 0.51–0.96; *p* = 0.01).	[107]
Pembrolizumab	Antiprogrammed cell death protein 1 antibody	In the KEYNOTE-040 study, pembrolizumab monotherapy prolonged overall survival in patients with advanced disease refractory to a platinum-based regimen (hazard ratio: 0.80; 95% confidence interval: 0.65–0.98; *p* = 0.0161). In the KEYNOTE-048 study, the addition of pembrolizumab to platinum-based chemotherapy in first-line therapy extended the overall survival of patients with PD-L1 positive disease at advanced stage (hazard ratio for CPS ≥ 20: 0.60; 95% confidence interval: 0.45–0.82; *p* = 0.0004; hazard ratio for CPS ≥ 1: 0.65; 95% confidence interval: 0.53–0.80; *p* < 0.0001).	[108,109]

## Data Availability

Not applicable.

## Abbreviations

CAF	cancer-associated fibroblast
CPS	combined positive score
CSF1	colony stimulating factor 1
CSF1R	colony stimulating factor 1 receptor
ECM	extracellular matrix
EGFR	epidermal growth factor receptor
EMT	epithelial–mesenchymal transition
EV	extracellular vesicle
FAP	fibroblast activation protein
HNC	head and neck cancer
HPV	human papillomavirus
ICI	immune checkpoint inhibitor
IFN	interferon
IL	interleukin
ISG15	interferon-stimulated gene 15
LC–MS	liquid chromatography–tandem mass spectrometry
MAPK	mitogen-activated protein kinase
MDSC	myeloid-derived suppressor cell
miRNA	microRNA
mtDNA	mitochondrial DNA
PD-1	programmed cell death 1
ROS	reactive oxygen species
SCC	squamous cell carcinoma
scRNA-seq	single-cell RNA-seq
SNP	single nucleotide polymorphism
TGF-β	transforming growth factor-β
TLR	toll-like receptor
TME	tumor microenvironment
TMEM	transmembrane protein
TNF-α	tumor necrosis factor-α
VEGF-C	vascular endothelial growth factor C

## References

[1] Dhanuthai K., Rojanawatsirivej S., Thosaporn W., Kintarak S., Subarnbhesaj A., Darling M., Kryshtalskyj E., Chiang C.P., Shin H.I., Choi S.Y. (2018). Oral cancer: A multicenter study. Med. Oral Patol. Oral Cir. Bucal.

[2] International Agency for Research on Cancer. http://gco.iarc.fr/.

[3] Cancer Registry Annual Report, 2016 (Taiwan). https://www.hpa.gov.tw/Pages/Detail.aspx?nodeid=269&pid=12235.

[4] Chuang S.L., Su W.W., Chen S.L., Yen A.M., Wang C.P., Fann J.C., Chiu S.Y., Lee Y.C., Chiu H.M., Chang D.C. (2017). Population-based screening program for reducing oral cancer mortality in 2,334,299 Taiwanese cigarette smokers and/or betel quid chewers. Cancer.

[5] Kerawala C., Roques T., Jeannon J.P., Bisase B. (2016). Oral cavity and lip cancer: United Kingdom National Multidisciplinary Guidelines. J. Laryngol. Otol..

[6] Montero P.H., Patel S.G. (2015). Cancer of the oral cavity. Surg. Oncol. Clin. N. Am..

[7] Mohan M., Jagannathan N. (2014). Oral field cancerization: An update on current concepts. Oncol. Rev..

[8] Hashim D., Genden E., Posner M., Hashibe M., Boffetta P. (2019). Head and neck cancer prevention: From primary prevention to impact of clinicians on reducing burden. Ann. Oncol..

[9] Koyfman S.A., Ismaila N., Crook D., D’Cruz A., Rodriguez C.P., Sher D.J., Silbermins D., Sturgis E.M., Tsue T.T., Weiss J. (2019). Management of the Neck in Squamous Cell Carcinoma of the Oral Cavity and Oropharynx: ASCO Clinical Practice Guideline. J. Clin. Oncol..

[10] Oosting S.F., Haddad R.I. (2019). Best Practice in Systemic Therapy for Head and Neck Squamous Cell Carcinoma. Front. Oncol..

[11] Sasco A.J., Secretan M.B., Straif K. (2004). Tobacco smoking and cancer: A brief review of recent epidemiological evidence. Lung Cancer.

[12] Warnakulasuriya K.A., Ralhan R. (2007). Clinical, pathological, cellular and molecular lesions caused by oral smokeless tobacco--a review. J. Oral Pathol. Med..

[13] Hernandez B.Y., Zhu X., Goodman M.T., Gatewood R., Mendiola P., Quinata K., Paulino Y.C. (2017). Betel nut chewing, oral premalignant lesions, and the oral microbiome. PLoS ONE.

[14] Li Y.C., Cheng A.J., Lee L.Y., Huang Y.C., Chang J.T. (2019). Multifaceted Mechanisms of Areca Nuts in Oral Carcinogenesis: The Molecular Pathology from Precancerous Condition to Malignant Transformation. J. Cancer.

[15] Lee C.H., Chang J.S., Syu S.H., Wong T.S., Chan J.Y., Tang Y.C., Yang Z.P., Yang W.C., Chen C.T., Lu S.C. (2015). IL-1β promotes malignant transformation and tumor aggressiveness in oral cancer. J. Cell Physiol..

[16] Baan R., Straif K., Grosse Y., Secretan B., El Ghissassi F., Bouvard V., Altieri A., Cogliano V. (2007). WHO International Agency for Research on Cancer Monograph Working Group. Carcinogenicity of alcoholic beverages. Lancet Oncol..

[17] Stornetta A., Guidolin V., Balbo S. (2018). Alcohol-Derived Acetaldehyde Exposure in the Oral Cavity. Cancers.

[18] Chen Y., Williams V., Filippova M., Filippov V., Duerksen-Hughes P. (2014). Viral carcinogenesis: Factors inducing DNA damage and virus integration. Cancers.

[19] Ha P.K., Califano J.A. (2004). The role of human papillomavirus in oral carcinogenesis. Crit. Rev. Oral Biol. Med..

[20] Hübbers C.U., Akgül B. (2015). HPV and cancer of the oral cavity. Virulence.

[21] Ali H., Donovan B., Wand H., Read T.R., Regan D.G., Grulich A.E., Fairley C.K., Guy R.J. (2013). Genital warts in young Australians five years into national human papillomavirus vaccination programme: National surveillance data. BMJ.

[22] Rosenquist K., Wennerberg J., Schildt E.B., Bladström A., Göran Hansson B., Andersson G. (2005). Oral status, oral infections and some lifestyle factors as risk factors for oral and oropharyngeal squamous cell carcinoma. A population-based case-control study in southern Sweden. Acta Otolaryngol..

[23] Slot D.E., Van der Weijden F., Ciancio S.G. (2014). Oral health, dental care and mouthwash associated with upper aerodigestive tract cancer risk in Europe: The ARCAGE study. Oral Oncol..

[24] Hashim D., Sartori S., Brennan P., Curado M.P., Wünsch-Filho V., Divaris K., Olshan A.F., Zevallos J.P., Winn D.M., Franceschi S. (2016). The role of oral hygiene in head and neck cancer: Results from International Head and Neck Cancer Epidemiology (INHANCE) consortium. Ann. Oncol..

[25] Gholizadeh P., Eslami H., Yousefi M., Asgharzadeh M., Aghazadeh M., Kafil H.S. (2016). Role of oral microbiome on oral cancers, a review. Biomed. Pharmacother..

[26] Karpiński T.M. (2019). Role of Oral Microbiota in Cancer Development. Microorganisms.

[27] Hsiao J.R., Chang C.C., Lee W.T., Huang C.C., Ou C.Y., Tsai S.T., Chen K.C., Huang J.S., Wong T.Y., Lai Y.H. (2018). The interplay between oral microbiome, lifestyle factors and genetic polymorphisms in the risk of oral squamous cell carcinoma. Carcinogenesis.

[28] Hu X., Zhang Q., Hua H., Chen F. (2016). Changes in the salivary microbiota of oral leukoplakia and oral cancer. Oral Oncol..

[29] Pushalkar S., Ji X., Li Y., Estilo C., Yegnanarayana R., Singh B., Li X., Saxena D. (2012). Comparison of oral microbiota in tumor and non-tumor tissues of patients with oral squamous cell carcinoma. BMC Microbiol..

[30] Lim Y., Fukuma N., Totsika M., Kenny L., Morrison M., Punyadeera C. (2018). The Performance of an Oral Microbiome Biomarker Panel in Predicting Oral Cavity and Oropharyngeal Cancers. Front. Cell Infect. Microbiol..

[31] Mager D.L., Haffajee A.D., Devlin P.M., Norris C.M., Posner M.R., Goodson J.M. (2005). The salivary microbiota as a diagnostic indicator of oral cancer: A descriptive, non-randomized study of cancer-free and oral squamous cell carcinoma subjects. J. Transl. Med..

[32] Yang C.Y., Yeh Y.M., Yu H.Y., Chin C.Y., Hsu C.W., Liu H., Huang P.J., Hu S.N., Liao C.T., Chang K.P. (2018). Oral Microbiota Community Dynamics Associated With Oral Squamous Cell Carcinoma Staging. Front. Microbiol..

[33] Katz J., Onate M.D., Pauley K.M., Bhattacharyya I., Cha S. (2011). Presence of Porphyromonas gingivalis in gingival squamous cell carcinoma. Int. J. Oral Sci..

[34] Ramachandran S., Ramadas K., Hariharan R., Rejnish Kumar R., Radhakrishna Pillai M. (2006). Single nucleotide polymorphisms of DNA repair genes XRCC1 and XPD and its molecular mapping in Indian oral cancer. Oral Oncol..

[35] Fan J., Liu W., Zhang M., Xing C. (2019). A literature review and systematic meta-analysis on XRCC3 Thr241Met polymorphism associating with susceptibility of oral cancer. Oncol. Lett..

[36] Xu C., Li C.Y., Kong A.N. (2005). Induction of phase I, II and III drug metabolism/transport by xenobiotics. Arch. Pharm. Res..

[37] Ghosh T., Gupta S., Bajpai P., Agarwal D., Agarwal M., Gupta O.P., Agrawal D. (2012). Association of CYP1A1, GSTM1, and GSTT1 gene polymorphism with risk of oral submucous fibrosis in a section of North Indian population. Mol. Biol. Rep..

[38] Agrawal D., Gupta S., Agarwal D., Gupta O.P., Agarwal M. (2010). Role of GSTM1 and GSTT1 polymorphism: Susceptibility to oral submucous fibrosis in the North Indian population. Oncology.

[39] Yadav B.K., Kaur J., Srivastava A., Ralhan R. (2009). Effect of polymorphisms in XRCC1, CCND1 and GSTM1 and tobacco exposure as risk modifier for oral leukoplakia. Int. J. Biol. Markers.

[40] Takeda K., Akira S. (2005). Toll-like receptors in innate immunity. Int. Immunol..

[41] Kluwe J., Mencin A., Schwabe R.F. (2009). Toll-like receptors, wound healing, and carcinogenesis. J. Mol. Med..

[42] Pisani L.P., Estadella D., Ribeiro D.A. (2017). The Role of Toll Like Receptors (TLRs) in Oral Carcinogenesis. Anticancer Res..

[43] Kauppila J.H., Mattila A.E., Karttunen T.J., Salo T. (2013). Toll-like receptor 5 and the emerging role of bacteria in carcinogenesis. Oncoimmunology.

[44] Chen F.C., Zhang F., Zhang Z.J., Meng S.Y., Wang Y., Xiang X.R., Wang C., Tang Y.Y. (2013). Tumor necrosis factor-α gene polymorphisms and risk of oral cancer: Evidence from a meta-analysis. Asian Pac. J. Cancer Prev..

[45] Li D., Hao S.H., Sun Y., Hu C.M., Ma Z.H., Wang Z.M., Liu J., Liu H.B., Ye M., Zhang Y.F. (2015). Functional Polymorphisms in COX-2 Gene Are Correlated with the Risk of Oral Cancer. Biomed. Res. Int..

[46] Yang W.H., Wang S.J., Chang Y.S., Su C.M., Yang S.F., Tang C.H. (2018). Association of Resistin Gene Polymorphisms with Oral Squamous Cell Carcinoma Progression and Development. Biomed. Res. Int..

[47] Hanahan D., Weinberg R.A. (2011). Hallmarks of cancer: The next generation. Cell.

[48] Mastronikolis N., Ragos V., Kyrodimos E., Chrysovergis A., Papanikolaou V., Mastronikolis S., Stamatelopoulos A., Tsiambas E. (2019). Mechanisms of C-myc oncogenic activity in head and neck squamous cell carcinoma. J. BUON.

[49] Marquard F.E., Jücker M. (2020). PI3K/AKT/mTOR signaling as a molecular target in head and neck cancer. Biochem. Pharmacol..

[50] Hsieh J.C., Wang H.M., Wu M.H., Chang K.P., Chang P.H., Liao C.T., Liau C.T. (2019). Review of emerging biomarkers in head and neck squamous cell carcinoma in the era of immunotherapy and targeted therapy. Head Neck..

[51] Picon H., Guddati A.K. (2020). Mechanisms of resistance in head and neck cancer. Am. J. Cancer Res..

[52] Barnes P., Yeboah F.A., Zhu J., Saahene R.O., Obirikorang C., Adinortey M.B., Amoani B., Kyei F., Akakpo P., Awuku Y.A. (2020). Prognostic Worth of Epidermal Growth Factor Receptor (EGFR) in Patients with Head and Neck Tumors. J. Cancer Epidemiol..

[53] Vermorken J.B., Mesia R., Rivera F., Remenar E., Kawecki A., Rottey S., Erfan J., Zabolotnyy D., Kienzer H.R., Cupissol D. (2008). Platinum-based chemotherapy plus cetuximab in head and neck cancer. N. Engl. J. Med..

[54] NCCN Guidelines for Head and Neck Cancers. https://www.nccn.org/professionals/physician_gls/pdf/head-and-neck.pdf.

[55] Zhu G., Pan C., Bei J.X., Li B., Liang C., Xu Y., Fu X. (2020). Mutant p53 in Cancer Progression and Targeted Therapies. Front. Oncol..

[56] Ragos V., Mastronikolis N.S., Tsiambas E., Baliou E., Mastronikolis S.N., Tsoukalas N., Patsouri E.E., Fotiades P.P. (2018). p53 mutations in oral cavity carcinoma. J. BUON.

[57] Stransky N., Egloff A.M., Tward A.D., Kostic A.D., Cibulskis K., Sivachenko A., Kryukov G.V., Lawrence M.S., Sougnez C., McKenna A. (2011). The mutational landscape of head and neck squamous cell carcinoma. Science.

[58] Lim A.M., Do H., Young R.J., Wong S.Q., Angel C., Collins M., Takano E.A., Corry J., Wiesenfeld D., Kleid S. (2014). Differential mechanisms of CDKN2A (p16) alteration in oral tongue squamous cell carcinomas and correlation with patient outcome. Int. J. Cancer.

[59] Adkins D., Ley J., Neupane P., Worden F., Sacco A.G., Palka K., Grilley-Olson J.E., Maggiore R., Salama N.N., Trinkaus K. (2019). Palbociclib and cetuximab in platinum-resistant and in cetuximab-resistant human papillomavirus-unrelated head and neck cancer: A multicentre, multigroup, phase 2 trial. Lancet Oncol..

[60] Massano J., Regateiro F.S., Januário G., Ferreira A. (2006). Oral squamous cell carcinoma: Review of prognostic and predictive factors. Oral Surg. Oral Med. Oral Pathol. Oral Radiol. Endod..

[61] Kapoor C., Vaidya S., Wadhwan V., Malik S. (2015). Lymph node metastasis: A bearing on prognosis in squamous cell carcinoma. Indian J. Cancer.

[62] Baik S.H., Seo J.W., Kim J.H., Lee S.K., Choi E.C., Kim J. (2019). Prognostic Value of Cervical Nodal Necrosis Observed in Preoperative CT and MRI of Patients With Tongue Squamous Cell Carcinoma and Cervical Node Metastases: A Retrospective Study. AJR Am. J. Roentgenol..

[63] Wissmann C., Detmar M. (2006). Pathways targeting tumor lymphangiogenesis. Clin. Cancer Res..

[64] Yanase M., Kato K., Yoshizawa K., Noguchi N., Kitahara H., Nakamura H. (2014). Prognostic value of vascular endothelial growth factors A and C in oral squamous cell carcinoma. J. Oral Pathol. Med..

[65] Naruse T., Yanamoto S., Yamada S.I., Takahashi H., Matsushita Y., Imayama N., Ikeda H., Shiraishi T., Fujita S., Ikeda T. (2015). Immunohistochemical study of vascular endothelial growth factor-C/vascular endothelial growth factor receptor-3 expression in oral tongue squamous cell carcinoma: Correlation with the induction of lymphangiogenesis. Oncol Lett..

[66] Sasahira T., Ueda N., Yamamoto K., Kurihara M., Matsushima S., Bhawal U.K., Kirita T., Kuniyasu H. (2014). Prox1 and FOXC2 act as regulators of lymphangiogenesis and angiogenesis in oral squamous cell carcinoma. PLoS ONE.

[67] Chen S., Chen L.H., Niu Y.H., Geng N.B., Feng C.J. (2020). AEG-1 promotes angiogenesis and may be a novel treatment target for tongue squamous cell carcinoma. Oral Dis..

[68] Ribatti D., Tamma R., Annese T. (2020). Epithelial-Mesenchymal Transition in Cancer: A Historical Overview. Transl. Oncol..

[69] Ota I., Masui T., Kurihara M., Yook J.I., Mikami S., Kimura T., Shimada K., Konishi N., Yane K., Yamanaka T. (2016). Snail-induced EMT promotes cancer stem cell-like properties in head and neck cancer cells. Oncol. Rep..

[70] Hsu D.S., Hwang W.L., Yuh C.H., Chu C.H., Ho Y.H., Chen P.B., Lin H.S., Lin H.K., Wu S.P., Lin C.Y. (2017). Lymphotoxin-beta interacts with methylated EGFR to mediate acquired resistance to cetuximab in head and neck cancer. Clin. Cancer Res..

[71] Goppel J., Mockelmann N., Munscher A., Sauter G., Schumacher U. (2017). Expression of epithelial-mesenchymal transition regulating transcription factors in head and neck squamous cell carcinomas. Anticancer Res..

[72] Zhou Y., Zhang H., Zhuo X., Liu Y., Zhang G., Tan Y. (2015). Over-expression of TWIST, an epithelial-mesenchymal transition inducer, predicts poor survival in patients with oral carcinoma. Int. J. Clin. Exp. Med..

[73] Seyedmajidi M., Seifi S., Moslemi D., Mozaffari S.F., Gholinia H., Zolfaghari Z. (2018). Immunohistochemical expression of TWIST in oral squamous cell carcinoma and its correlation with clinicopathologic factors. J. Cancer Res. Ther..

[74] Bai Y., Sha J., Kanno T. (2020). The Role of Carcinogenesis-Related Biomarkers in the Wnt Pathway and Their Effects on Epithelial-Mesenchymal Transition (EMT) in Oral Squamous Cell Carcinoma. Cancers.

[75] Reyes M., Flores T., Betancur D., Peña-Oyarzún D., Torres V.A. (2020). Wnt/β-Catenin Signaling in Oral Carcinogenesis. Int. J. Mol. Sci..

[76] Mikels A.J., Nusse R. (2006). Wnts as ligands: Processing, secretion and reception. Oncogene.

[77] Wang S.H., Chang J.S., Hsiao J.R., Yen Y.C., Jiang S.S., Liu S.H., Chen Y.L., Shen Y.Y., Chang J.Y., Chen Y.W. (2017). Tumour cell-derived WNT5B modulates in vitro lymphangiogenesis via induction of partial endothelial-mesenchymal transition of lymphatic endothelial cells. Oncogene.

[78] Chen Y.L., Wu W.L., Jang C.W., Yen Y.C., Wang S.H., Tsai F.Y., Shen Y.Y., Chen Y.W. (2019). Interferon-stimulated gene 15 modulates cell migration by interacting with Rac1 and contributes to lymph node metastasis of oral squamous cell carcinoma cells. Oncogene.

[79] Cajee U.F., Hull R., Ntwasa M. (2012). Modification by ubiquitin-like proteins: Significance in apoptosis and autophagy pathways. Int. J. Mol. Sci..

[80] Kazanietz M.G., Caloca M.J. (2017). The Rac GTPase in Cancer: From Old Concepts to New Paradigms. Cancer Res..

[81] Choi D., Spinelli C., Montermini L., Rak J. (2019). Oncogenic Regulation of Extracellular Vesicle Proteome and Heterogeneity. Proteomics.

[82] Schubert A., Boutros M. (2021). Extracellular vesicles and oncogenic signaling. Mol. Oncol..

[83] Wang S.H., Liou G.G., Liu S.H., Chang J.S., Hsiao J.R., Yen Y.C., Chen Y.L., Wu W.L., Chang J.Y., Chen Y.W. (2019). Laminin γ2-enriched extracellular vesicles of oral squamous cell carcinoma cells enhance in vitro lymphangiogenesis via integrin α3-dependent uptake by lymphatic endothelial cells. Int. J. Cancer.

[84] Rupaimoole R., Slack F.J. (2017). MicroRNA therapeutics: Towards a new era for the management of cancer and other diseases. Nat. Rev. Drug Discov..

[85] Anastasiadou E., Jacob L.S., Slack F.J. (2018). Non coding RNA networks in cancer. Nat. Rev. Cancer.

[86] Gomes C.C., Gomez R.S. (2008). MicroRNA and oral cancer: Future perspectives. Oral Oncol..

[87] Min A., Zhu C., Peng S., Rajthala S., Costea D.E., Sapkota D. (2015). MicroRNAs as Important Players and Biomarkers in Oral Carcinogenesis. Biomed. Res. Int..

[88] Fang C., Li Y. (2019). Prospective applications of microRNAs in oral cancer. Oncol. Lett..

[89] Shiah S.G., Hsiao J.R., Chang W.M., Chen Y.W., Jin Y.T., Wong T.Y., Huang J.S., Tsai S.T., Hsu Y.M., Chou S.T. (2014). Downregulated miR329 and miR410 promote the proliferation and invasion of oral squamous cell carcinoma by targeting Wnt-7b. Cancer Res..

[90] Cheng C.M., Shiah S.G., Huang C.C., Hsiao J.-R., Chang J.-Y. (2016). Up-regulation of miR-455-5p by the TGF-β-SMAD signalling axis promotes the proliferation of oral squamous cancer cells by targeting, U.B.E.2.B. J. Pathol..

[91] Chang W.M., Lin Y.F., Su C.Y., Peng H.Y., Chang Y.C., Lai T.C., Wu G.H., Hsu Y.M., Chi L.H., Hsiao J.R. (2016). Dysregulation of RUNX2/Activin-A Axis upon miR-376c Downregulation Promotes Lymph Node Metastasis in Head and Neck Squamous Cell Carcinoma. Cancer Res..

[92] Hsing E.W., Shiah S.G., Peng H.Y., Chen Y.W., Chuu C.P., Hsiao J.R., Lyu P.C., Chang J.Y. (2019). TNF-α-induced miR-450a mediates TMEM182 expression to promote oral squamous cell carcinoma motility. PLoS ONE.

[93] Zhao M., Mishra L., Deng C.X. (2018). The role of TGF-β/SMAD4 signaling in cancer. Int. J. Biol. Sci..

[94] Pang X., Tang Y.L., Liang X.H. (2018). Transforming growth factor-β signaling in head and neck squamous cell carcinoma: Insights into cellular responses. Oncol. Lett..

[95] Yoshida C.A., Yamamoto H., Fujita T., Furuichi T., Ito K., Inoue K., Yamana K., Zanma A., Takada K., Ito Y. (2004). Runx2 and Runx3 are essential for chondrocyte maturation, and Runx2 regulates limb growth through induction of Indian hedgehog. Genes Dev..

[96] Wajant H. (2009). The role of TNF in cancer. Results Probl. Cell Differ..

[97] Josephs S.F., Ichim T.E., Prince S.M., Kesari S., Marincola F.M., Escobedo A.R., Jafri A. (2018). Unleashing endogenous TNF-alpha as a cancer immunotherapeutic. J. Transl. Med..

[98] Wrzesiński T., Szelag M., Cieślikowski W.A., Ida A., Giles R., Zodro E., Szumska J., Poźniak J., Kwias Z., Bluyssen H.A. (2015). Expression of pre-selected TMEMs with predicted ER localization as potential classifiers of ccRCC tumors. BMC Cancer.

[99] Cheng Z., Guo J., Chen L., Luo N., Yang W., Qu X. (2015). Overexpression of TMEM158 contributes to ovarian carcinogenesis. J. Exp. Clin. Cancer Res..

[100] Zhao L.C., Shen B.Y., Deng X.X., Chen H., Zhu Z.G., Peng C.H. (2016). TMEM45B promotes proliferation, invasion and migration and inhibits apoptosis in pancreatic cancer cells. Mol. Biosyst..

[101] Boxberg M., Leising L., Steiger K., Jesinghaus M., Alkhamas A., Mielke M., Pfarr N., Götz C., Wolff K.D., Weichert W. (2019). Composition and Clinical Impact of the Immunologic Tumor Microenvironment in Oral Squamous Cell Carcinoma. J. Immunol..

[102] Peltanova B., Raudenska M., Masarik M. (2019). Effect of tumor microenvironment on pathogenesis of the head and neck squamous cell carcinoma: A systematic review. Mol. Cancer.

[103] Mohan S.P., Bhaskaran M.K., George A.L., Thirutheri A., Somasundaran M., Pavithran A. (2019). Immunotherapy in Oral Cancer. J. Pharm. Bioallied. Sci..

[104] Kujan O., van Schaijik B., Farah C.S. (2020). Immune Checkpoint Inhibitors in Oral Cavity Squamous Cell Carcinoma and Oral Potentially Malignant Disorders: A Systematic Review. Cancers.

[105] Jiang Y., Chen M., Nie H., Yuan Y. (2019). PD-1 and PD-L1 in cancer immunotherapy: Clinical implications and future considerations. Hum. Vaccin Immunother..

[106] Zhang J.Y., Yan Y.Y., Li J.J., Adhikari R., Fu L.W. (2020). PD-1/PD-L1 Based Combinational Cancer Therapy: Icing on the Cake. Front. Pharmacol..

[107] Ferris R.L., Blumenschein G., Fayette J., Guigay J., Colevas A.D., Licitra L., Harrington K., Kasper S., Vokes E.E., Even C. (2016). Nivolumab for recurrent squamous-cell carcinoma of the head and neck. N. Engl. J. Med..

[108] Cohen E.E.W., Soulieres D., Le Tourneau C., Dinis J., Licitra L., Ahn M.J., Soria A., Machiels J.P., Mach N., Mehra R. (2019). Pembrolizumab versus methotrexate, docetaxel, or cetuximab for recurrent or metastatic head-and-neck squamous cell carcinoma (KEYNOTE-040): A randomised, open-label, phase 3 study. Lancet.

[109] Burtness B., Harrington K.J., Greil R., Soulières D., Tahara M., de Castro G., Psyrri A., Basté N., Neupane P., Bratland Å. (2019). Pembrolizumab alone or with chemotherapy versus cetuximab with chemotherapy for recurrent or metastatic squamous cell carcinoma of the head and neck (KEYNOTE-048): A randomised, open-label, phase 3 study. Lancet.

[110] Zandberg D.P., Strome S.E. (2014). The role of the PD-L1: PD-1 pathway in squamous cell carcinoma of the head and neck. Oral Oncol..

[111] Ren D., Hua Y., Yu B., Ye X., He Z., Li C., Wang J., Mo Y., Wei X., Chen Y. (2020). Predictive biomarkers and mechanisms underlying resistance to PD1/PD-L1 blockade cancer immunotherapy. Mol. Cancer..

[112] Chen Y.P., Wang Y.Q., Lv J.W., Li Y.Q., Chua M.L.K., Le Q.T., Lee N., Colevas A.D., Seiwert T., Hayes D.N. (2019). Identification and validation of novel microenvironment-based immune molecular subgroups of head and neck squamous cell carcinoma: Implications for immunotherapy. Ann. Oncol..

[113] Qin X., Yan M., Wang X., Xu Q., Wang X., Zhu X., Shi J., Li Z., Zhang J., Chen W. (2018). Cancer-associated Fibroblast-derived IL-6 Promotes Head and Neck Cancer Progression via the Osteopontin-NF-kappa B Signaling Pathway. Theranostics.

[114] Tsai M.S., Chen W.C., Lu C.H., Chen M.F. (2019). The prognosis of head and neck squamous cell carcinoma related to immunosuppressive tumor microenvironment regulated by IL-6 signaling. Oral Oncol..

[115] Johnson D.E., O’Keefe R.A., Grandis J.R. (2018). Targeting the IL-6/JAK/STAT3 signalling axis in cancer. Nat. Rev. Clin. Oncol..

[116] Punyani S.R., Sathawane R.S. (2013). Salivary level of interleukin-8 in oral precancer and oral squamous cell carcinoma. Clin. Oral Investig..

[117] Nishio Y., Gojoubori T., Kaneko Y., Shimizu N., Asano M. (2015). Cancer cell-derived IL-8 induces monocytic THP1 cells to secrete IL-8 via the mitogen-activated protein kinase pathway. Tumour Biol..

[118] Chuang C.-Y., Sung W.-W., Wang L., Lin W.L., Yeh K.T., Su M.C., Hsin C.H., Lee S.Y., Wu B.C., Cheng Y.W. (2012). Differential impact of IL-10 expression on survival and relapse between HPV16-positive and -negative oral squamous cell carcinomas. PLoS ONE.

[119] Wang S., Sun M., Gu C., Wang X., Wang X., Chen D., Zhao E., Jiao X., Zheng J. (2014). Expression of CD163, interleukin-10, and interferon-gamma in oral squamous cell carcinoma: Mutual relationships and prognostic implications. Eur. J. Oral Sci..

[120] Gonçalves A.S., Arantes D.A., Bernardes V.F., Jaeger F., Silva J.M., Silva T.A., Aguiar M.C., Batista C. (2015). Immunosuppressive mediators of oral squamous cell carcinoma in tumour samples and saliva. Hum. Immunol..

[121] Watari K., Shibata T., Kawahara A., Sata K., Nabeshima H., Shinoda A., Abe H., Azuma K., Murakami Y., Izumi H. (2014). Tumor-derived interleukin-1 promotes lymphangiogenesis and lymph node metastasis through M2-type macrophages. PLoS ONE.

[122] Shchors K., Shchors E., Rostker F., Lawlor E.R., Brown-Swigart L., Evan G.I. (2006). The Myc-dependent angiogenic switch in tumors is mediated by interleukin 1beta. Genes Dev..

[123] Huang Y.H., Chang C.Y., Kuo Y.Z., Fang W.Y., Kao H.Y., Tsai S.T., Wu L.W. (2019). Cancer-associated fibroblast-derived interleukin-1β activates protumor C-C motif chemokine ligand 22 signaling in head and neck cancer. Cancer Sci..

[124] Astradsson T., Sellberg F., Berglund D., Ehrsson Y.T., Laurell G.F.E. (2019). Systemic Inflammatory Reaction in Patients with Head and Neck Cancer-An Explorative Study. Front. Oncol..

[125] Allison K.E., Coomber B.L., Bridle B.W. (2017). Metabolic reprogramming in the tumour microenvironment: A hallmark shared by cancer cells and T lymphocytes. Immunology.

[126] Cerezo M., Rocchi S. (2020). Cancer cell metabolic reprogramming: A keystone for the response to immunotherapy. Cell Death Dis..

[127] Kumar D., New J., Vishwakarma V., Joshi R., Enders J., Lin F., Dasari S., Gutierrez W.R., Leef G., Ponnurangam S. (2018). Cancer-Associated Fibroblasts Drive Glycolysis in a Targetable Signaling Loop Implicated in Head and Neck Squamous Cell Carcinoma Progression. Cancer Res..

[128] Colegio O.R., Chu N.Q., Szabo A.L., Chu T., Rhebergen A.M., Jairam V., Cyrus N., Brokowski C.E., Eisenbarth S.C., Phillips G.M. (2014). Functional polarization of tumour-associated macrophages by tumour-derived lactic acid. Nature.

[129] Jha A.K., Huang S.C., Sergushichev A., Lampropoulou V., Ivanova Y., Loginicheva E., Chmielewski K., Stewart K.M., Ashall J., Everts B. (2015). Network integration of parallel metabolic and transcriptional data reveals metabolic modules that regulate macrophage polarization. Immunity.

[130] El Kasmi K.C., Stenmark K.R. (2015). Contribution of metabolic reprogramming to macrophage plasticity and function. Semin Immunol..

[131] Cemerski S., Cantagrel A., Van Meerwijk J.P., Romagnoli P. (2002). Reactive oxygen species differentially affect T cell receptor-signaling pathways. J. Biol. Chem..

[132] Chamoto K., Chowdhury P.S., Kumar A., Sonomura K., Matsuda F., Fagarasan S., Honjo T. (2017). Mitochondrial activation chemicals synergize with surface receptor PD-1 blockade for T cell-dependent antitumor activity. Proc. Natl. Acad. Sci. USA.

[133] Maj T., Wang W., Crespo J., Zhang H., Wang W., Wei S., Zhao L., Vatan L., Shao I., Szeliga W. (2017). Oxidative stress controls regulatory T cell apoptosis and suppressor activity and PD-L1-blockade resistance in tumor. Nat. Immunol..

[134] Sung Y.J., Kao T.Y., Kuo C.L., Fan C.C., Cheng A.N., Fang W.C., Chou H.Y., Lo Y.K., Chen C.H., Jiang S.S. (2018). Mitochondrial Lon sequesters and stabilizes p53 in the matrix to restrain apoptosis under oxidative stress via its chaperone activity. Cell Death Dis..

[135] Voos W., Pollecker K. (2020). The Mitochondrial Lon Protease: Novel Functions off the Beaten Track?. Biomolecules.

[136] Kuo C.L., Chou H.Y., Chiu Y.C., Cheng A.N., Fan C.C., Chang Y.N., Chen C.H., Jiang S.S., Chen N.J., Lee A.Y. (2020). Mitochondrial oxidative stress by Lon-PYCR1 maintains an immunosuppressive tumor microenvironment that promotes cancer progression and metastasis. Cancer Lett..

[137] Cheng A.N., Cheng L.C., Kuo C.L., Lo Y.K., Chou H.Y., Chen C.H., Wang Y.H., Chuang T.H., Cheng S.J., Lee A.Y. (2020). Mitochondrial Lon-induced mtDNA leakage contributes to PD-L1-mediated immunoescape via STING-IFN signaling and extracellular vesicles. J. Immunother. Cancer..

[138] Qi Z., Barrett T., Parikh A.S., Tirosh I., Puram S.V. (2019). Single-cell sequencing and its applications in head and neck cancer. Oral Oncol..

[139] Huang L.Y., Hsieh Y.P., Wang Y.Y., Hwang D.Y., Jiang S.S., Huang W.T., Chiang W.F., Liu K.J., Huang T.T. (2020). Single-Cell Analysis of Different Stages of Oral Cancer Carcinogenesis in a Mouse Model. Int. J. Mol. Sci..

[140] Evrard D., Szturz P., Tijeras-Raballand A., Astorgues-Xerri L., Abitbol C., Paradis V., Raymond E., Albert S., Barry B., Faivre S. (2019). Macrophages in the microenvironment of head and neck cancer: Potential targets for cancer therapy. Oral Oncol..

[141] Pathria P., Louis T.L., Varner J.A. (2019). Targeting Tumor-Associated Macrophages in Cancer. Trends Immunol..

[142] Cohen E.E.W., Nabell L., Wong D.J.L., Day T.A., Daniels G.A., Milhem M.M., Deva S., Jameson M.B., Guntinas-Lichius O., Almubarak M. (2019). Phase 1b/2, open label, multicenter study of intratumoral SD-101 in combination with pembrolizumab in anti-PD-1 treatment naïve patients with recurrent or metastatic head and neck squamous cell carcinoma (HNSCC). J. Clin. Oncol..

[143] Chen X., Song E. (2019). Turning foes to friends: Targeting cancer-associated fibroblasts. Nat. Rev. Drug Discov..

[144] Huber M.A., Kraut N., Park J.E., Schubert R.D., Rettig W.J., Peter R.U., Garin-Chesa P. (2003). Fibroblast activation protein: Differential expression and serine protease activity in reactive stromal fibroblasts of melanocytic skin tumors. J. Investig. Dermatol..

[145] Soerensen M.M., Ros W., Rodriguez-Ruiz M.E., Robbrecht D., Rohrberg K., Martin-Liberal J., Lassen U., Bermejo I.M., Lolkema M.P., Tabernero J. (2018). Safety, PK/PD, and anti-tumor activity of RO6874281, an engineered variant of interleukin-2 (IL-2v) targeted to tumor-associated fibroblasts via binding to fibroblast activation protein (FAP). J. Clin. Oncol..

